# Event extraction across multiple levels of biological organization

**DOI:** 10.1093/bioinformatics/bts407

**Published:** 2012-09-03

**Authors:** Sampo Pyysalo, Tomoko Ohta, Makoto Miwa, Han-Cheol Cho, Jun'ichi Tsujii, Sophia Ananiadou

**Affiliations:** ^1^ National Centre for Text Mining and School of Computer Science, University of Manchester, Manchester, UK; ^2^Department of Computer Science, University of Tokyo, Tokyo, Japan; ^3^Microsoft Research Asia, Beijing, China

## Abstract

**Motivation:** Event extraction using expressive structured representations has been a significant focus of recent efforts in biomedical information extraction. However, event extraction resources and methods have so far focused almost exclusively on molecular-level entities and processes, limiting their applicability.

**Results:** We extend the event extraction approach to biomedical information extraction to encompass all levels of biological organization from the molecular to the whole organism. We present the ontological foundations, target types and guidelines for entity and event annotation and introduce the new multi-level event extraction (MLEE) corpus, manually annotated using a structured representation for event extraction. We further adapt and evaluate named entity and event extraction methods for the new task, demonstrating that both can be achieved with performance broadly comparable with that for established molecular entity and event extraction tasks.

**Availability:** The resources and methods introduced in this study are available from http://nactem.ac.uk/MLEE/.

**Contact:** pyysalos@cs.man.ac.uk

**Supplementary information:** Supplementary data are available at *Bioinformatics* online.

## 1 INTRODUCTION

A detailed understanding of biological systems requires the ability to trace cause and effect across multiple levels of biological organization, from molecular-level reactions to cellular, tissue- and organ-level effects to organism-level outcomes (Kitano, 2002). Consequently, any effort aiming to comprehensively represent biological systems must address entities and processes at all of these levels.

This challenge has so far been only partially met in biomedical information extraction (IE) and text mining, which aim to improve access to domain knowledge by automating aspects of processing the literature. Until recently, efforts in domain IE were primarily focused on the basic task of recognizing mentions of relevant entities such as genes and proteins in text (Yeh *et al.*, 2005) and on the extraction of pairwise relations between these representing, for example, protein–protein interactions (Krallinger *et al.*, 2007; Nédellec, 2005). Such representations lack the capacity to capture any but the simplest of associations.

**Fig. 1.**
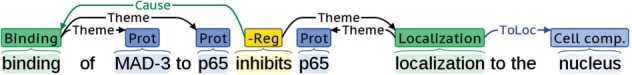
Example sentence with event annotation. Prot, -Reg and Cell comp. abbreviated for Protein, Negative regulation and Cell component, respectively

In recent years, there has been increasing interest in the extraction of structured representations capable of capturing associations of arbitrary numbers of participants in specific roles. Such approaches to IE, frequently termed *event extraction*, are capable of representing complex associations—such as the binding of a protein to another inhibiting its localization to a specific cellular compartment (Fig. 1)—and open many new opportunities for domain text mining applications ranging from semantic search to database and pathway curation support (Ananiadou *et al.*, 2010). There is significant momentum behind the move to richer representations for IE: more than 30 groups have introduced methods for biomedical event extraction in shared tasks (Kim *et al.*, 2011a, b); event-annotated corpora have been introduced for many extraction targets, including DNA methylation (Ohta *et al.*, 2011a), protein modifications (Pyysalo *et al.*, 2011) and the molecular mechanisms of infectious diseases (Pyysalo *et al.*, 2012c); event extraction methods have been applied to automatically analyze all 20 million PubMed abstracts (Björne *et al.*, 2010); and event extraction analyses are being integrated into literature search systems such as MEDIE^1^ and applied in support of advanced tasks such as pathway curation (Ohta *et al.*, 2011b).

While the event extraction approach has been demonstrated to be applicable to a variety of extraction targets across different subdomains of biomedical science, related efforts all share a key restriction: nearly exclusive focus on molecular-level entities and events.^2^ Entities such as proteins and genes and events such as binding and phosphorylation are an important part of the picture of biological systems, but still only a part, and any IE approach aiming to capture the whole picture must also consider other levels of biological organization.

In this study, our aim is to extend the scope of existing event extraction resources and methods to levels of biological organization ranging from the subcellular to the organism level as a step toward developing the capacity for the automatic extraction of these targets from the entire available literature. Toward this end, we propose relevant entity and event types for annotation across these levels with reference to community-standard ontologies, develop a set of detailed guidelines for their annotation in text and create structured event annotation marking over 8000 entities and 6000 events in abstracts relevant to cancer biology, previously annotated by domain experts to identify spans of text relevant to their interests. Using this data, we perform experiments using state-of-the-art methods for both entity mention detection and event extraction to analyze the feasibility of extraction using existing tools, further evaluating the benefits of specific adaptations of such tools to the novel task.

## 2 APPROACH

### 2.1 Corpus texts and reference annotation

We selected as the starting point for our study a recently introduced corpus of 262 PubMed abstracts on angiogenesis, the development of new blood vessels from existing ones. The domain involves a tissue/organ-level process that is closely associated with cancer and other organism-level pathologies and whose molecular basis is increasingly understood (Carmeliet and Jain, 2000), and domain texts thus represent a good test case for structured IE across multiple levels of biological organization.

The corpus texts were previously annotated by Wang *et al.* (2011) using a typed-span representation, marking references to molecular level entities, cells, tissues and domain-relevant processes. We use these annotations created by domain experts as a reference for identifying statements of interest for our annotation, which focuses on introducing structured event annotation and solidifying the ontological basis of the existing entity annotation.

### 2.2 Representation

We apply the specific event representation first formalized in the BioNLP 2009 Shared Task on event extraction and applied in numerous resources and methods introduced since. In this representation, Entity mentions (or *entities*, for short) are marked as continuous spans of text identified with a type (e.g. Protein), and event structures (or *events*) are *n*-ary associations of participants—entities or other events—each of which is identified as participating in the event in a specific role (e.g. *Theme* and *Cause*). Each event is assigned a type from a fixed set defined for the task (e.g. Binding and Phosphorylation) and is associated with a specific span of text stating the event, termed the *event trigger*. Events can additionally be marked with *modifiers* identifying the event as being, e.g. explicitly negated, or stated in a speculative context. We refer to (Kim *et al.*, 2011a) for a detailed presentation of the representation.

**Fig. 2.**
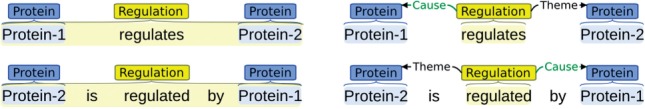
Span versus structure. Although a representation using nested, typed spans (left) can capture the fact that specific entities participate in a process, it lacks the mechanisms to express, e.g. the direction of causality. The structured event representation (right) differentiates *Themes* from *Causes*

Given the starting point of the existing corpus annotations, our event annotation effort proceeds from *spans* to a *structured* representation that can represent complex associations between arbitrary numbers of entities (Fig. 1) and many other aspects that the typed-span representation cannot, such as the direction of causality (Fig. 2).

In addition to selecting the general form of representation, to define a specific event annotation scheme, we must also fix the annotated entity and event types as well as the roles, participant scopes and modifiers applied. For these, we build on previously introduced resources targeting the molecular level, basing our extensions on domain ontologies.

### 2.3 Ontological basis

We take as basic the division between continuants (or endurants) and occurrents (perdurants, processes or events) (see e.g. Smith, 2003) and adopt the general principle followed also in major previously introduced event-annotated resources that references to continuants such as material entities are annotated using the entity representation and references to occurrents such as biological processes are annotated as events.^3^

In the definition of our annotation scheme, we aim for compatibility with existing event-annotated corpora—primarily the five ‘main task’ corpora introduced in the BioNLP Shared Tasks—to allow these to be used together with the annotations that we create and to assure that our extensions are coherent with existing resources derived from these corpora. Thus, for molecular-level entity and process types, we adopt the scope, semantics and annotation guidelines of these resources as closely as possible without compromising coverage of mentions marked as relevant by domain experts. For entities and processes not in scope of previous event resources, we propose new types for annotation, basing type and scope definitions and annotation guidelines on major community-curated ontological resources from the open biomedical ontologies (OBO) foundry^4^ (Smith *et al.*, 2007). In brief, before primary annotation, we analyzed mentions marked in the reference annotation to identify entity and process types not in scope of previously defined event annotation guidelines and then defined new types and guidelines for annotation with reference to selected ontologies. These are summarized in the following.

### 2.4 Annotation scheme

The focus our extensions of previously proposed event annotation schemes is on *anatomical entities* such as cells, tissues and organs and processes involving them such as growth, remodeling and death.^5^

For anatomical entity types, we adopt a top-level division by granularity (Kumar *et al.*, 2004) based primarily on the upper-level structure of the Common Anatomy Reference Ontology (CARO) (Haendel *et al.*, 2008), an organism-independent ontology of anatomy based on the human-specific Foundational Model of Anatomy (Rosse and Mejino, 2003 , 2008), as outlined in our previous work on anatomical entities (Pyysalo *et al.*, 2012b). To account for pathological anatomy-level entities (e.g. glioma)—out of scope of ontologies of canonical anatomy—we draw on the approach proposed by (Smith *et al.*, 2005). Table 1 summarizes the primary entity types applied in the annotation.^6^

**Table 1.** Primary entity types, related ontology terms and annotation counts

Type	Term(s)	Examples	Count
Organism			
Organism*^a^*	Single cell org._caro_, multi-cellular org._caro_	*Human, mice, C. albicans*	722
Anatomy			
Organism subdivision	Organism subdivision _caro_	*Head, thorax, hindlimb, legs*	49
Anatomical system	Anatomical system_caro_	*Central nervous system, pulmonary system*	18
Organ	Compound organ_caro_	*Heart, eyes, skin*	176
Multi-tissue structure	Multi-tissue structure_caro_	*Blood vessel, peritoneal membrane, lymph nodes*	514
Tissue	Portion of tissue_caro_	*Endothelium, adipose tissue, capillary*	426
Cell	Cell_cl_	*Endothelial cells, HUVECs, pericyte, cancer cells*	1198
Cellular component	Cellular component_go_	*Nuclei, focal adhesions, extracellular matrix*	145
Developing anatomical structure	Developing anatomical structure_ehdaa_	*Embryo*	6
Organism substance	Portion of organism substance_caro_	*Blood, serum, plasma, urine*	142
Immaterial anatomical entity	Immaterial anatomical entity_caro_	*Lumen, preperitoneal space, marrow cavity*	15
Pathological formation	Cancer_doid_, benign neoplasm_doid_	*Tumor, colorectal cancer, gliomas*	910
Molecule			
Drug or compound*^a^*	Inorganic molecular entity_chebi_, drug_chebi_	*Oxygen, ethanol, bevacizumab, thalidomide*	944
Gene or gene product*^a^*	Gene_so_, RNA_chebi_, protein_chebi_	*VEGF, p53, IL-8, endostatin, thrombin*	2962

Labels in gray identify informal categories used in evaluation.

*^a^* Annotated also in previously introduced event extraction resources. t_o_ identifies a term t in an ontology o; ontology identifiers are OBO Foundry prefixes (namespaces).

**Fig. 3.**
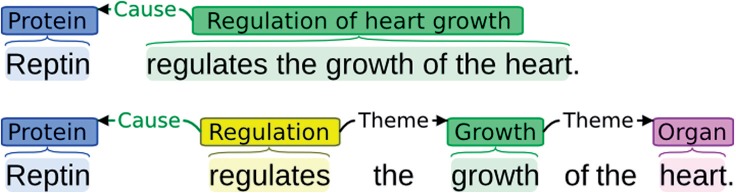
Annotation with detailed GO terms (top; hypothetical) and event annotation with general types (bottom; applied)

For event types, we draw primarily on the biological process subontology of the gene ontology (GO) (Ashburner *et al.*, 2000). As in previous event-annotated resources, we consider only general upper-level GO terms such as growth_GO_: references to specific processes included in GO through composite terms such as regulation of heart growth_GO_ are captured using the explicitly structured representation^7^ (Fig. 3). We also capture general statements of causal association using Regulation types, as in previous event annotation efforts (see e.g. Kim *et al.*, 2008). Following the scope of the reference annotation, we introduce event annotation also for intentionally planned processes (e.g. injection) as outlined in the Ontology for Biomedical Investigations (OBI) (Brinkman *et al.*, 2010), using a single, non-specific type Planned process for their annotation. We additionally introduce a Breakdown event for annotating pathological processes that result in the breakdown of anatomical structures. Finally, we apply the domain-specific Blood vessel development type to annotate references to blood vessel development through expressions such as ‘angiogenesis’ that incorporate both the process and the affected entity. Expressions such as ‘blood vessel development’ that allow explicitly structured annotation are marked with a separate entity annotation (e.g. ‘blood vessel’) and an event (e.g. ‘development’) taking the entity as its Theme. The primary event types are summarized in Table 2.

**Table 2.** Primary event types, argument roles, related ontology terms and annotation counts

Type	Arguments	Term(s)	Examples	Count
Anatomical				
Cell proliferation	*Theme*	Cell proliferation_go_	*proliferating [ECs], [MCs] accumulated*	133
Development	*Theme*	Developmental process_go_	*[skin] development, [stress fiber] formation*	316
Blood vessel development	*Theme, At-Loc*	Blood vessel development_go_	*angiogenesis, neovascularization*	855
Growth	*Theme*	Growth_go_	*growth [of arteries], [tumour] growth*	169
Death	*Theme*	Death_go_	*[connective tissue] necrosis, [cell] apoptosis*	97
Breakdown	*Theme*	—	*[ECM] degradation, damage [to tumor cell]*	69
Remodeling	*Theme*	Tissue remodeling_go_	*[vascular] remodeling, changes [in membrane]*	33
Molecular				
Synthesis	*Theme*	Biosynthetic process_go_	*[ATP] synthesis, production [of NOS]*	17
Gene expression*^a^*	*Theme*	Gene expression_go_	*expression [of VEGF]*	435
Transcription*^a^*	*Theme*	Transcription, DNA-dependent_go_	*[VEGF] mRNA expression*	37
Catabolism*^a^*	*Theme*	Catabolic process_go_	*[p53] breakdown*	26
Phosphorylation*^a^*	*Theme, Site*	Phosphorylation_go_	*phosphorylation [of KDR]*	33
Dephosphorylation*^a^*	*Theme, Site*	Dephosphorylation_go_	*[Mcl-1] dephosphorylation*	6
General				
Localization*^a^*	*Theme, At/From/To-Loc*	Localization_go_	*[VEGF] colocalized, [VPF was] secreted*	450
Binding*^a^*	*Theme, Site*	Binding_go_, biological adhesion_go_	*[cell] adhesion, [GDP-]bound [Rab5a]*	184
Regulation*^a^*	*Theme, Cause, Site*	Biological regulation_go_	*[aMSH] modulates [activation of AP-1]*	773
Positive regulation*^a^*	*Theme, Cause, Site*	Pos.regulation of biol.proc. _go_	*[insulin] stimulates [VEGF expression]*	1327
Negative regulation*^a^*	*Theme, Cause, Site*	Neg.regulation of biol.proc. _go_	*Inhibition [of NO synthase by L-NAME]*	921
Planned				
Planned process	*Theme, Instrument*	Planned process_obi_	*injection [of U-995], [UFT] administration*	643

Labels in gray identify categories used in evaluation: events of the Anatomical category involve Organism or Anatomy entities (Table 1); Molecular involve Molecule entities; others can involve any entity type.

*^a^*Annotated also in previously introduced event extraction resources.

For event participants, we apply otherwise standard roles included also in previous efforts (e.g. Theme and Cause) but introduce the role Instrument for distinguishing entities used to carry out planned processes from those that undergo the effects of the process.^8^ Also as in previously introduced event corpora, we apply two binary modifiers, Negation and Speculation, marking events as explicitly negated (e.g. ‘cells did not proliferate’) or stated in a speculative context (e.g. ‘growth might be inhibited’), respectively.

We refer to the detailed annotation guidelines (Pyysalo *et al.*, 2012a) for specifics of the annotation, but note here one systematic difference between our annotation and the scope of the reference ontologies: the ontologies define idealized types—canonical anatomy and physiological processes—but texts primarily refer to real-world instances that do not fill these exacting criteria (Bada and Hunter, 2011). We thus interpreted the scope of mentions marked with a specific type to include not only the corresponding (canonical) types defined in ontologies but also variants such as entities or processes influenced by mutation, including also pathological variants. As specific examples, we mark ‘*cancer cell* as Cell’, and ‘*[cancer] growth*’ as Growth.

### 2.5 Annotation process

Primary annotation was performed by a PhD biologist with more than a decade of experience in text annotation who had previously coordinated several event annotation efforts (TO). Annotations were made using the brat rapid annotation tool (Stenetorp *et al.*, 2012).

Detailed annotation guidelines were prepared based on those for the GENIA and BioNLP Shared Task guidelines and refined throughout annotation to clarify ambiguous cases and document specific decisions made in annotation. We refer to the supplementary documentation and these guidelines (Pyysalo *et al.*, 2012a) for further details of the annotation scheme and the detailed definitions of all annotated types.

## 3 METHODS

This section presents the automatic entity mention detection and event extraction methods applied in this study, their adaptation to the novel extraction targets and the experimental setup.

Following standard practice in domain event extraction studies, we divide the automatic extraction task into two separate stages, the detection of entity mentions and the extraction of events involving these and evaluate system performance on these two separately.

### 3.1 Entity mention detection

For entity mention detection experiments, we applied NERsuite, a named entity recognition toolkit based on the CRFsuite implementation (Okazaki, 2007) of conditional random fields (Lafferty *et al.*, 2001). NERsuite is capable of efficiently incorporating features based on token matching against large-scale lexical resources, and the applied version achieves an *F* score of 86.4% on the BioCreative II evaluation standard (GENETAG) (Tanabe *et al.*, 2005), effectively matching the performance of the best available systems for the task.^9^

Following initial sentence splitting and tokenization, we perform lemmatization, POS-tagging and shallow parsing using the GENIA tagger (Tsuruoka and Tsujii, 2005). Next, we optionally perform a matching step using dictionaries compiled from the UMLS Metathesaurus (Bodenreider, 2004), Entrez Gene (Maglott *et al.*, 2005) and OBO Foundry (Smith *et al.*, 2007) resources. We then extract a comprehensive set of features for machine learning, building on orthographic, lexical, syntactic and dictionary match information (see Supplementary information).

Following preliminary development test experiments, we chose to apply a single model that jointly predicts all entity types. In the final experiments, we compare a *base* model using only from the newly annotated data without external resources with a *dictionary*-supported model that incorporates features from matching against the lexical resources derived from UMLS, Entrez Gene and OBO foundry ontologies.

### 3.2 Event extraction

For event extraction, we applied EventMine,^10^ a pipeline-based event extraction system using support vector machines (SVM). EventMine takes as input document text and entity annotations, and extracts event structures and modifications. EventMine outperforms the best systems participating in the original BioNLP Shared Task 2011 on the GE and ID data sets (with *F* scores 58.0% and 57.6%, respectively) and is competitive with the best systems on the EPI data set (Kim *et al.*, 2011b; Miwa *et al.*, 2012).

EventMine consists of four modules: (i) event trigger detection marks likely triggers and assigns them types, (ii) argument detection identifies likely trigger-argument pairs and assigns them roles, (iii) multi-argument event detection combines trigger-argument pairs into likely event structures and (iv) modification detection assigns modification flags (*Negation* and *Speculation*). Each module addresses its task as a multi-label classification problem, using the one-versus-rest SVM implementation of (Fan *et al.*, 2008), with a rich feature set generated from tokens and paths in the predicate-argument structure analyses of the Enju parser (Miyao *et al.*, 2009) and the dependency analyses of the GDep parser (Sagae and Tsujii, 2007). In feature generation, EventMine applies semantic class generalization—e.g. merging Positive regulation and Regulation types for some features—to reduce the data sparsity and the number of different classes in the classification problems. In addition to training EventMine on the newly introduced corpus, we also introduced a set of generalization rules appropriate to the introduced types. We refer to supplementary documentation and (Miwa *et al.*, 2012) for further details on EventMine.

We performed event extraction experiments in two settings: training only on the newly introduced data (*base* model) and training using *stacking*, incorporating predictions from a model trained on the BioNLP Shared Task 2011 GE data set (Kim *et al.*, 2011b) as the source corpus. No other external resources were used in the evaluation.

### 3.3 Experimental setup

The annotated data were initially divided into training, development and test sets. The test set was held out during method development and parameter selection. For the final experiment, methods were trained on the combination of training and development data and evaluated on the test set.

We evaluate both entity mention detection and event extraction performance using the standard precision, recall and *F* score^11^ metrics, microaveraged over instance-level true-positive, false-positive and false-negative counts.

For entity mention detection, we apply the evaluation protocol and tools of the BioNLP/JNLPBA shared task 2004 (Kim *et al.*, 2004), evaluating results using three matching criteria: exact span match, left boundary match and right boundary match. The first requires the extent of a predicted entity mention to be identical to that of a gold mention for the prediction to be considered correct, whereas the latter two only require one of the boundaries defining the extent to match. We require the type of the predicted and annotated entities to be identical in all cases.

For event extraction, we adapt the evaluation protocol and tools introduced in the BioNLP Shared Task 2011 (Kim *et al.*, 2011a), including providing gold entity annotations as given for event extraction. We apply the primary matching criteria defined in the task, which otherwise require event structures to be identical but include the *approximate span* and *approximate recursive* relaxations to exact match: the former allows small variation in predicted event trigger spans and the latter permits differences in the secondary arguments of recursive event structures for matches. For detailed definitions, we refer to (Kim *et al.*, 2011a).

## 4 RESULTS AND DISCUSSION

We next present the primary results of the annotation effort and the entity mention detection and event extraction experiments.

### 4.1 Annotation effort and results

We estimate the concentrated effort to produce the corpus annotation to have totalled approximately 250 hours, of which approximately 100 hours used on guideline development, management and annotation consistency checking. The effort required to produce structured event annotation is thus broadly comparable to the initial effort by domain experts to mark text spans of interest (Wang *et al.*, 2011).

**Fig. 4.**
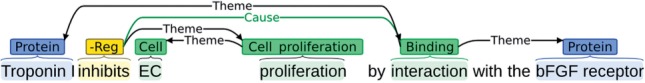
Example Negative regulation (-Reg) event connecting entities at different levels of biological organization

Table 3 presents the overall statistics of the annotated multilevel event extraction (MLEE) corpus. We note that the texts include comparable numbers of molecular and anatomy-level entity mentions, with a lower but still notable number of organism mentions. The event counts show a higher density of anatomical than molecular-level events, although general biological events dominate overall. Overall, 1222 events, or 18% of the total, involve either directly or indirectly (through participating events) arguments at both the molecular and anatomy levels (Fig. 4). Table 4 presents corpus statistics with reference to those for the three largest event-annotated corpora in the recent BioNLP shared task 2011. We note that although the MLEE corpus is smaller than these corpora focusing on the molecular level in terms of e.g. word count, there is less difference in the number of entity annotations, and the MLEE corpus has more event annotations than two of the shared task corpora. The introduced corpus thus has a very high density of event annotations, which we attribute in part to the novel entity and event types allowing a more comprehensive representation of statements in text.

**Table 3.** Overall corpus statistics

Item	Train	Devel	Test	Total
Document	131	44	87	262
Sentence	1271	457	880	2608
Word	27 875	9610	19 103	56 588
Entity	4147	1431	2713	8291
Organism	359	126	237	722
Anatomy	1844	589	1166	3599
Molecule	1944	716	1310	3970
Event	3296	1175	2206	6677
Anatomical	810	269	596	1675
Molecular	340	125	240	705
General	1851	627	1176	3654
Planned	295	154	194	643

See Tables 1 and 2 for entity and event categories.

**Table 4.** Comparison of corpus statistics with BioNLP Shared Task 2011 corpora annotated using the same representation

Item	MLEE	EPI	GE	ID
Document	262	1200	1224	30*^a^*
Word	56 588	253 628	348 908	153 153
Entity	8291	15190	21616	12740
Event	6677	3714	24967	4150

*^a^* The ID document count is low as the corpus consists of full-text documents, not abstracts.

We refer to Supplementary Material Section 1.3 for an evaluation of the corpus annotation consistency.

### 4.2 Entity mention detection

The overall evaluation results for entity mention detection are listed in Table 5. We find a consistent benefit from the use of the lexical resources, with e.g. a 3.6% point improvement in *F* score (15% reduction in error) for strict matching. As expected, evaluated performance is notably higher under the relaxed criteria, in particular for right boundary matching. This suggests comparatively many errors in the choice of noun premodifiers included in annotation span, a distinction that may not be of critical importance for many applications.

**Table 5.** Overall entity mention detection results (prec/rec/*F* score)

Model	Exact	Matching criterion	
		Left boundary	Right boundary
Base	77.03 / 69.18 / 72.89	79.85 / 71.72 / 75.57	82.47 / 74.07 / 78.04
Dictionary	79.49 / 73.77 / 76.52	82.59 / 76.64 / 79.50	84.68 / 78.58 / 81.52

Table 6 lists a breakdown of performance by entity category for the dictionary model. The detection of Organism mentions is most reliable despite their sparseness in the data, conforming to previous results indicating this entity class to represent a comparatively easy problem (Gerner *et al.*, 2010). The detection of mentions of entities of the Anatomy and Molecule categories can be performed at broadly comparable accuracy on this corpus containing balanced numbers of annotations of the two, suggesting that fine-grained anatomical entity detection is no more difficult than established molecular level entity detection tasks.

**Table 6.** Entity mention detection results by category for dictionary model (prec/rec/*F* score)

Category	Exact	Matching criterion	
		Left boundary	Right boundary
Organism	90.82 / 82.10 / 86.24	91.79 / 82.97 / 87.16	91.79 / 82.97 / 87.16
Anatomy	77.47 / 72.70 / 75.01	78.67 / 73.83 / 76.17	84.58 / 79.38 / 81.90
Molecule	79.37 / 73.25 / 76.18	84.54 / 78.03 / 81.15	83.54 / 77.10 / 80.19

The overall entity mention detection performance, approaching or exceeding 80% in *F* score depending on evaluation criteria, is a very promising result given the novelty of the task and its many challenging aspects, most obviously that it involves more than 10 distinct entity types. As points of comparison, the best single system at the well-established single-class BioCreative 2 Gene Mention task achieved an *F* score of 87.2% under matching criteria that in cases accept more than one specific span as correct (Wilbur *et al.*, 2007) and the highest-performing system at the original BioNLP/JNLPBA shared task, involving the detection of entities of five different types, achieved an *F* score of 72.6% under the exact matching criterion (Kim *et al.*, 2004).

### 4.3 Event extraction

The overall results for event extraction using EventMine are presented in Table 7. The results demonstrate that the stacked model incorporating information from the previously introduced GE corpus outperforms a purely corpus-internal model. Although the improvement from incorporating the independently annotated out-of-domain data is somewhat modest, the result does indicate that the annotation has met its aim to maintain compatibility with this key resource for molecular-level event annotation.

**Table 7.** Overall event extraction results

Model	Prec	Rec	*F* score
Base	56.53	48.72	52.34
Stacking (GE)	56.38	50.77	53.43

As for entity mention detection, performance for the best model, at over 50% *F* score for event extraction, is very promising for a first experiment on the new task. For reference, the best results in the recent, widely attended BioNLP Shared Task 2011 for the same evaluation criteria were 56.0% *F* score for the GE task, 53.3% *F* score for the EPI task and 55.6% *F* score for the ID task (Table 4) (Kim *et al.*, 2011b). Reaching this general level of performance suggests that the task is feasible for current event extraction technology and that the annotation consistency and the size of the introduced corpus are sufficient for reliable extraction.

Table 8 gives a breakdown of the event extraction performance by category. Interestingly, we find that events involving anatomical entities are more reliably extracted than those involving molecular-level ones, despite the model incorporating information from a corpus with a larger number of molecular level event annotations than the total number of annotations in the MLEE corpus. This is a very encouraging finding for event extraction for anatomical processes, indicating that the representation and extraction methods are well suited for the task.

**Table 8.** Event extraction results by category for stacked model

Category	Prec	Rec	*F* score
Anatomical	80.91	72.05	76.22
Molecular	68.44	75.63	71.86
General	43.87	38.99	41.29
Planned	56.68	51.96	54.22
Modification	47.95	29.92	36.85

Event categories as defined in Table 2; *Modification* gives performance for *Negation* and *Speculation* detection.

## 5 CONCLUSION

We have presented the MLEE corpus, a resource aiming to extend the coverage of resources and methods for structured event extraction from the molecular level to encompass all levels from the subcellular to the organism. Experiments using state-of-the-art entity mention detection and event extraction methods demonstrated that the newly proposed extraction targets can be met with reasonable performance using the MLEE corpus, with approximately 80% overall *F* score for entity mention detection and over 50% *F* score for event extraction using standard evaluation criteria.

In future work, we will focus on the extension of the annotations and extraction methods to improve the domain independence of our annotation to allow the application of the introduced extraction methods at large scale to automatically annotate the entire available literature. The results of these extraction efforts will be made available through search systems such as MEDIE to further improve access to the biomedical literature by facilitating structured semantic queries across multiple levels of biological organization, for example to find statements regarding the inhibition of organ growth by specific molecular-level entities or events.

All resources introduced in this study, including the annotated corpus, guidelines, the evaluation tools and the methods are available from http://nactem.ac.uk/MLEE/.

## Footnotes

^1^http://www.nactem.ac.uk/medie/.

^2^Some recent tasks have considered also organisms (primarily unicellular, see e.g. Bossy *et al.*, 2012; Pyysalo *et al.*, 2012c), but prior event extraction efforts have not specifically targeted entities and processes between the molecular and organism levels.

^3^We use the terms ‘entity’ and ‘event’ primarily following usage in IE, to identify forms of representation, not ontological categories. In particular, the latter term does not denote a category distinct from processes.

^4^http://obofoundry.org.

^5^Although the existing corpus annotation of (Wang *et al.*, 2011) identifies such mentions, they are typed nonspecifically, using e.g. Positive regulation to mark ‘*development*’ and Negative regulation for ‘*[cell] death*’.

^6^Note that we differentiate between types applied in annotation and their (broadly) corresponding ontology types.

^7^This annotation strategy can be viewed as partly analogous to efforts to make GO term structure explicit (Mungall *et al.*, 2011).

^8^For example in ‘*rats were injected with hyperforin*’, the Organism mention (‘*rats*’) is the *Theme* of the Planned process (‘*injected*’) and the Drug or compound mention (‘*hyperforin*’) is the *Instrument*.

^9^http://nersuite.nlplab.org

^10^http://www.nactem.ac.uk/EventMine/

^11^Specifically  where *p* is precision and *r* recall.
